# Improved Cell-Penetrating Zinc-Finger Nuclease Proteins for Precision Genome Engineering

**DOI:** 10.1038/mtna.2015.6

**Published:** 2015-03-10

**Authors:** Jia Liu, Thomas Gaj, Mark C Wallen, Carlos F Barbas

**Affiliations:** 1 The Skaggs Institute for Chemical Biology, The Scripps Research Institute, La Jolla, California, USA; 2 Department of Chemistry, The Scripps Research Institute, La Jolla, California, USA; 3 Department of Cell and Molecular Biology, The Scripps Research Institute, La Jolla, California, USA; 4 Shanghai Institute for Advanced Immunochemical Studies (SIAIS), ShanghaiTech University, Shanghai, China; 5 Deceased

**Keywords:** genome editing, protein delivery, zinc-finger nuclease

## Abstract

Safe, efficient, and broadly applicable methods for delivering site-specific nucleases into cells are needed in order for targeted genome editing to reach its full potential for basic research and medicine. We previously reported that zinc-finger nuclease (ZFN) proteins have the innate capacity to cross cell membranes and induce genome modification via their direct application to human cells. Here, we show that incorporation of tandem nuclear localization signal (NLS) repeats into the ZFN protein backbone enhances cell permeability nearly 13-fold and that single administration of multi-NLS ZFN proteins leads to genome modification rates of up to 26% in CD4^+^ T cells and 17% in CD34^+^ hematopoietic stem/progenitor cells. In addition, we show that multi-NLS ZFN proteins attenuate off-target effects and that codelivery of ZFN protein pairs facilitates dual gene modification frequencies of 20–30% in CD4^+^ T cells. These results illustrate the applicability of ZFN protein delivery for precision genome engineering.

The emergence of site-specific DNA endonucleases and genome editing has empowered researchers with the unprecedented ability to manipulate virtually any gene across a broad spectrum of cell types and organisms.^1,2,3^ The technologies most commonly used to achieve this—homing endonucleases,^4,5^ zinc-finger nucleases (ZFNs),^6,7,8^ TAL effector nucleases (TALENs),^9,10,11^ and CRISPR/Cas9^12,13,14^—have the potential to revolutionize basic biological research and transform medicine, as evidenced by recent clinical trials for HIV/AIDS based on ZFN-mediated modification of the HIV-1 coreceptor chemokine (C-C motif) receptor 5 (CCR5).^15^ These systems are generally configured to induce targeted DNA double-strand breaks (DSBs) that stimulate the cellular DNA repair machinery, typically leading to one of two outcomes: gene knockout^16^ via mutagenic nonhomologous end joining (NHEJ) or, in the presence of an accompanying donor template, targeted gene integration^17^ or correction^8,18^ by homology-directed repair (HDR). While recent technological advances have helped make genome editing a routine endeavor for nonspecialized laboratories, establishing safe and efficient delivery methods for sensitive applications remains challenging. Current methods for achieving this are based on transfection or electroporation^19^ of nuclease-encoded DNA or mRNA or the use of viral vector delivery systems.^20^ These strategies, however, are limited by a number of factors: viral vectors are time-consuming to produce, capable of chromosomal integration, hampered by repetitive elements (*e.g.*, TAL effector repeats)^21,22,23^ and, in the case of adenoassociated virus (AAV), size-constrained. Nonviral delivery systems, on the other hand, are restricted to certain cell types and have been reported to show toxicity from electroporation^24^ or transfection.^25^ In addition, regardless of the delivery strategy used, high levels of nuclease expression from DNA could lead to increased off-target activity,^26^ a detrimental side effect that might have serious consequences for therapeutic genome editing. Indeed, in order for nuclease-mediated genome engineering to reach its full potential, new methods that improve their specificity and safety are needed.

The direct delivery of nuclease proteins into mammalian cells has been shown to be a viable alternative to DNA or mRNA-based delivery systems. Unlike methods that rely on expression from nucleic acids, protein delivery poses no risk for chromosomal insertions and only exposes the cell to the nuclease for a short period of time, thereby reducing off-target effects.^27^ To date, nuclease proteins have been administered directly into cells via cell-penetrating peptides,^28,29,30^ ligand-mediated endocytosis,^31^ electroporation,^32,33^ or transfection^34^ and retro-^35^ or lentivirus particles.^36,37,38^ In particular, we previously showed that ZFN proteins are inherently cell-permeable and capable of inducing genome modifications via their direct application to human cells.^27^ ZFNs are chimeric endonucleases consisting of the cleavage domain of the FokI restriction endonuclease and a custom-designed Cys_2_-His_2_ zinc-finger DNA-binding protein,^39,40^ the latter of which possesses the innate ability to cross cell membranes likely due to its high overall positive charge. Due to its simplicity and ability to improve the specificity of genome editing, ZFN protein delivery represents a promising approach for modifying cells for *ex vivo* genome engineering applications. However, for most cell-types, consecutive protein treatments are necessary to achieve high levels of genomic modification, a drawback that limits the scope and scalability of this methodology.

Here, we explore the use of nuclear localization signals (NLS)—highly positively charged peptide domains that have the innate ability to cross cell membranes—as a means to enhance ZFN protein cell permeability. We demonstrate that incorporation of tandem NLS repeats into the ZFN protein backbone enhances ZFN cell-penetrating activity and leads to highly efficient genome modification in a diverse range of cell types, including primary CD4^+^ T cells, CD34^+^ hematopoietic stem/progenitor cells (HSPCs) and induced pluripotent stem cells (iPSCs). In addition, we show that multi-NLS ZFN proteins retain the ability to mitigate off-target effects and mediate high levels of dual gene modification in CD4^+^ T cells, illustrating the potential of ZFN protein delivery for *ex vivo* genome engineering processes.

## Results

### Improving ZFN protein delivery via tandem NLS repeats

As a means to enhance the innate cell-penetrating activity of ZFN proteins, we explored the possibility of genetically fusing protein transduction domains (PTDs) to the N-terminus of ZFNs. We^27^ and others^29^ previously reported that incorporation of the cell-penetrating peptide sequence from the HIV-1 TAT protein^41^ or the poly-Arg peptide^42^ impairs ZFN protein expression. We thus expanded the scope of this approach by separately incorporating two additional PTDs, penetratin^43^ and transportan,^44^ into the ZFN protein backbone. While both fusion proteins could be expressed in yields sufficient for downstream analysis (**Supplementary Figure S1**), reduced activity was observed for both proteins *in vitro* and no improvement in genomic modification was evident for either ZFN protein in cell culture (**Supplementary Figure S2**).

**Figure 1:**
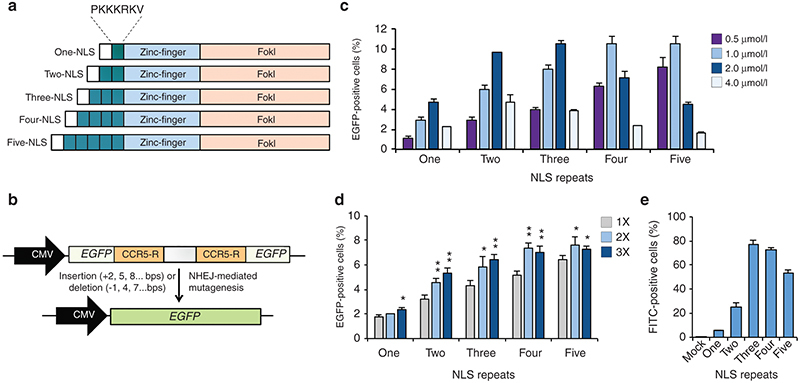
Tandem NLS repeats enhance ZFN protein activity. (**a**) Diagrams of one- to five-NLS ZFN proteins. Green and white boxes indicate NLS and poly-His domains, respectively. (**b**) Schematic representation of the HEK293 EGFP reporter system used to evaluate multi-NLS ZFN protein activity. “CCR5-R” indicates the “right” CCR5 ZFN protein binding sites. (**c**) Percentage of EGFP-positive reporter cells measured by flow cytometry following treatment with increasing concentrations of one- to five-NLS ZFN protein. (**d**) Percentage of EGFP-positive reporter cells measured by flow cytometry following one to three consecutive treatments with 0.5 μmol/l one- to five-NLS ZFN protein. (**e**) Percentage of FITC-positive HEK293 cells measured by flow cytometry following treatment with 1 μmol/l fluorescein-conjugated one- to five-NLS ZFN proteins for 1 hour. “Mock” indicates cells treated with serum-free medium. Bars represent ± SD (*n* = 3). *P < 0.05; **P < 0.01; ***P < 0.001 by *t*-test.

ZFNs typically contain a single N-terminal Simian vacuolating virus 40 (SV40) NLS sequence (PKKKRKV) that mediates nuclear import but does not measurably contribute to its intrinsic cell-penetrating activity.^27^ Because in some contexts NLS sequences possess an innate ability to cross cell membranes^45^ and mediate protein transfection,^46^ we hypothesized that tandem NLS repeats could enhance ZFN protein cell-permeability. To test this, we fused one, two, three, or four additional repeats of the SV40 NLS to the N-terminus of ZFN proteins that already contained one NLS and were designed to target the human *CCR5* gene (**Figure 1a**).^47^ We generated ZFN proteins in high yield (>2 mg/l) and >80% purity from the soluble fraction of *Escherichia coli* lysates but observed varying levels of proteolysis of three-, four- and five-NLS ZFN proteins (**Supplementary Figure S3**). Compared to native one-NLS ZFN protein, only four- and five-NLS proteins showed a decrease in cleavage activity *in vitro* (**Supplementary Figure S3**). In particular, low-levels of nonspecific cleavage were evident for the five-NLS ZFN proteins (**Supplementary Figure S3**), likely due to nonspecific association between the highly positively charged N-terminus of the ZFN protein and the DNA backbone.

We evaluated the ability of these multi-NLS ZFN proteins to enter cells and stimulate mutagenesis using a previously described human embryonic kidney (HEK) 293 reporter cell line (**Figure 1b**).^27,48^ This system features an integrated EGFP gene whose expression has been disabled by the presence of a frame-shift mutation introduced by a ZFN cleavage site that contains two symmetrical binding sites for the “right” CCR5 ZFN protein. Due to the stochastic nature of NHEJ, approximately one-third of all ZFN-induced DSBs can restore the EGFP reading frame. Thus, the ability of ZFN protein to penetrate cells is correlated with the percentage of EGFP positive cells measured by flow cytometry. Direct application of multi-NLS ZFN protein to reporter cells resulted in a two- to sevenfold increase in EGFP fluorescence compared to the native one-NLS ZFN protein (**Figure 1c**). We observed maximum activity (~11% EGFP-positive cells) after a single treatment with 1 μmol/l four-NLS ZFN protein and >8% EGFP-positive cells after a single treatment with 0.5 μmol/l five-NLS ZFN protein (**Figure 1c**). In contrast, a single treatment with 0.5 μmol/l one-NLS ZFN protein led to ~1% EGFP positive cells (**Figure 1c**), while Lipofectamine-mediated transient transfection of ZFN expression vector resulted in ~6% EGFP positive cells (**Supplementary Figure S4**). Consecutive protein administrations increased the percentage of EGFP positive cells, with repeated treatment of 0.5 μmol/l two-, three-, and four-NLS ZFN protein leading to a ~1.5-fold increase in EGFP fluorescence (**Figure 1d**). This effect, however, plateaued after two treatments with four- and five-NLS protein. Collectively, these results indicate that genetic incorporation of tandem NLS repeats is an effective approach for enhancing ZFN protein activity.

### Tandem NLS repeats enhance ZFN cell-permeability

We next set out to determine whether tandem NLS repeats enhanced ZFN protein cell-permeability. We incubated HEK293 cells with 1 μmol/l one-, two-, three-, four-, and five-NLS ZFN proteins labeled with fluorescein-5-maleimide, which reacts with the Cys residue present on the surface of the FokI cleavage domain.^27^ ZFN proteins with no NLS were not included in this analysis since these proteins were previously shown to exhibit cell-penetrating activity equivalent to one-NLS proteins.^27^ After incubation, cells were washed three consecutive times with heparin to remove nonspecific surface-bound protein and ZFN internalization was measured by flow cytometry. Depending on the number of NLS repeats, we observed between 4- and 13-fold increases in cellular fluorescence compared to single-NLS ZFN protein (**Figure 1e**). Notably, three- and four-NLS proteins showed the greatest increase in cell permeability, indicating that tandem NLS repeats enhance ZFN protein uptake. Although no appreciable increase in EGFP fluorescence was observed among cells transfected with multi-NLS ZFNs (**Supplementary Figure S4**)—indicating perhaps a neutral role for tandem NLS repeats with respect to nuclear transport—additional studies are needed to determine this in context of ZFN protein delivery.

### Efficient modification of endogenous genes via direct delivery of multi-NLS ZFN proteins

**Figure 2:**
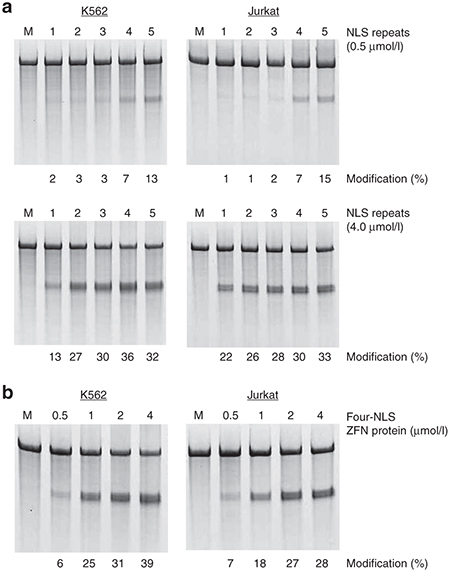
Modification of endogenous genes by direct delivery of multi-NLS ZFN proteins. (**a**,**b**) Frequency of endogenous *CCR5* gene modification in K562 and Jurkat cells treated with (**a**) 0.5 or 4 μmol/l one- to five-NLS ZFN proteins targeting the *CCR5* gene or (**b**) increasing concentrations of four-NLS ZFN proteins targeting the *CCR5* gene. Modification determined by the Surveyor nuclease assay. “M” indicates cells treated with serum-free medium.

**Figure 3:**
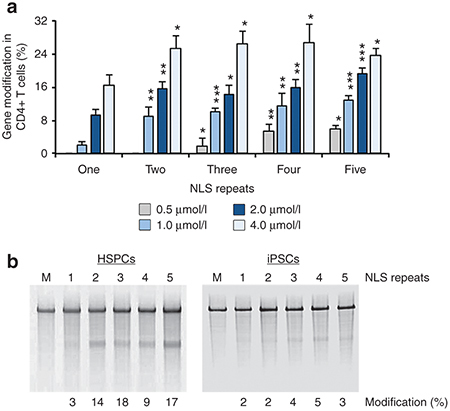
Modification of endogenous genes in primary cells and stem cells by direct delivery of multi-NLS ZFN proteins. (**a**,**b**) Frequency of endogenous *CCR5* gene modification in (**a**) stimulated human CD4^+^ T cell treated with increasing amounts of one- to five-NLS ZFN proteins targeting the *CCR5* gene or (**b**, left) hematopoietic stem/progenitor cells (HSPCs) and (**b**, right) induced pluripotent stem cells (iPSCs) treated with 4.0 μmol/l native (one-NLS) or multi-NLS ZFN proteins targeting the *CCR5* gene. “M” indicates cells treated with serum-free medium. Gene modification determined by the Surveyor nuclease assay. Bars represent ± SD (*n* = 3). *P < 0.05, **P < 0.01, ***P < 0.001 by *t*-test.

We next evaluated whether NLS repeats enhanced the efficiency of endogenous gene modifications induced by ZFN proteins. To test this, K562 and Jurkat cells were treated once with 0.5, 1, 2, and 4 μmol/l one-, two-, three-, four-, and five-NLS ZFN proteins that targeted the *CCR5* gene. Treatments were performed with equimolar amounts of “left” and “right” ZFN proteins with identical NLS repeats and the frequency of endogenous gene modification was evaluated using the Surveyor nuclease assay.^49^ Compared to one-NLS ZFN proteins, we observed up to 15-fold increases in gene modification for each multi-NLS protein tested (**Figure 2a**). In particular, analysis of DNA isolated from K562 and Jurkat cells treated with 4 μmol/l four- and five-NLS ZFN proteins revealed *CCR5* modification rates of 36% and 33%, respectively. Gene mutagenesis was dose-dependent and typically plateaued with four-NLS proteins at high (*i.e.*, 4 μmol/l) protein concentrations in both cell types (**Figure 2b**). We also tested the ability of multi-NLS ZFN proteins to mediate gene modification in several therapeutically relevant, but traditionally difficult-to-transfect, primary cell types including CD4^+^ T cells,^47,50,51^ CD34^+^ hematopoietic stem/progenitor cells (HSPCs)^52,53,54^ and induced pluripotent stem cells (iPSCs).^55^ Maximum activity was ~26% in stimulated CD4^+^ T cells (**Figure 3a**), 17% in HSPCs (**Figure 3b**), and 5% in iPSCs (**Figure 3b**). Modification of the *CCR5* gene in CD4^+^ T cells was dose-dependent, but saturated with three-NLS ZFN proteins at high protein concentrations. Sequence analysis of cloned *CCR5* alleles amplified from each treated cell type confirmed the presence of ZFN-induced insertions and deletions (**Supplementary Figure S5**).

**Figure 4:**
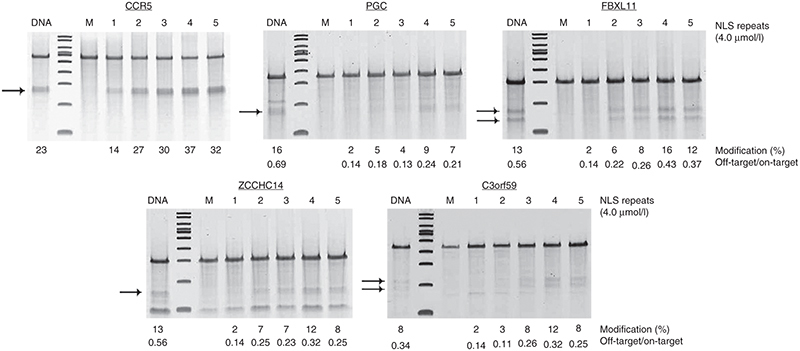
Cleavage specificity of multi-NLS ZFN proteins. Surveyor nuclease analysis of the *CCR5*, *PGC*, *FBXL11*, *ZCCHC14*, and *C3orf59* loci in K562 cells treated with 4 μmol/l one- to five-NLS ZFN proteins targeting the *CCR5* gene. Gene modification and the ratio between off-target to on-target cleavage are denoted. “DNA” indicates cells nucleofected with 1 μg each of the “left” and “right” CCR5 one-NLS ZFN expression vectors. “M” indicates cells treated with serum-free medium. Arrows indicate anticipated cleavage products.

**Figure 5:**
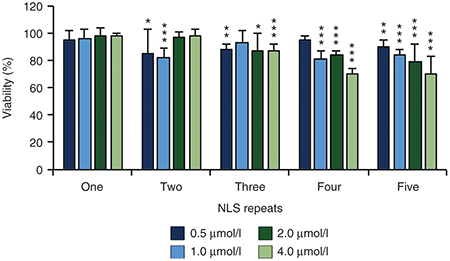
Toxicity induced by multi-NLS ZFN proteins. Viability of stimulated CD4^+^ T cells treated once with increasing concentrations of one- to five-NLS ZFN proteins targeting the *CCR5* gene. Proliferation was measured 2 days after protein treatments. Data normalized to cells treated with serum-free medium. Bars represent ± SEM (*n* = 3). *P < 0.05; **P < 0.01; ***P < 0.001 by *t*-test.

To investigate the cleavage specificity of multi-NLS ZFN proteins, we measured the activity of the CCR5 multi-NLS ZFN proteins at four previously described off-target cleavage sites in K562 cells.^56^ Each multi-NLS ZFN protein showed reduced off-target activity compared to ZFNs expressed from plasmid DNA delivered into cells via nucleofection (**Figure 4**). At two off-target cleavage sites, *PGC* and *C3orf59*, two-NLS ZFN proteins showed specificity comparable to one-NLS proteins; however, an increase in off-target modifications was observed for three-, four-, and five-NLS proteins in comparison to the one-NLS ZFN proteins (**Figure 4**). In order to evaluate whether directly delivered multi-NLS ZFN proteins induced toxicity, we treated stimulated CD4^+^ T cells with increasing concentrations of one-, two-, three-, four-, and five-NLS ZFN proteins and measured cell viability using the MTT/XTT assay, which utilizes a quantitative, colorimetric readout to assess the metabolic activity of cells. We observed >80% viability in CD4^+^ T cells treated with two- and three-NLS proteins but modest toxicity (~70% viability) for cells incubated with high doses (4 μmol/l) of four- and five-NLS proteins (**Figure 5**). Collectively, these findings indicate that multi-NLS ZFN proteins facilitate high levels of endogenous gene modification in a broad range of human cell types with minimal adverse effects.

### Modification of the *CCR5* and *CXCR4* genes in CD4^+^ T cells by multi-NLS ZFN proteins

**Figure 6:**
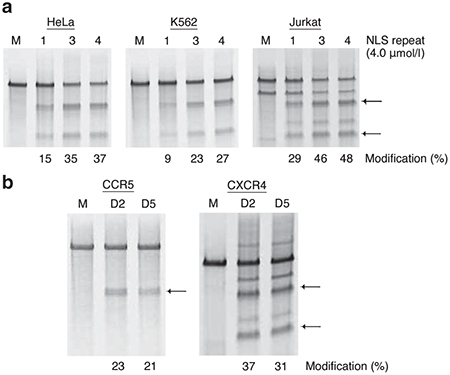
Modification of the *CCR5* and *CXCR4* genes in CD4^+^ T cells by direct delivery of multi-NLS ZFN protein pairs. (**a**) Frequency of endogenous *CXCR4* gene modification in HeLa, K562, and Jurkat cells treated with 4 μmol/l one-, three-, and four-NLS ZFN proteins targeting the *CXCR4* gene. (**b**) Frequency of endogenous *CCR5* and *CXCR4* gene modification in stimulated CD4^+^ T cells treated simultaneously with 2 μmol/l each three-NLS ZFN proteins targeting the *CCR5* and *CXCR4* genes. Gene modification was determined by Surveyor nuclease assay. Arrows indicate expected cleavage products. “M” indicates cells treated with serum-free medium and “D2” and “D5” indicate genomic DNA isolated from CD4^+^ T cells at 2 and 5 days after ZFN protein treatments, respectively.

Finally, we sought to evaluate the generality and potential of multi-NLS ZFN protein delivery for inducing dual gene modifications in CD4^+^ T cells. Past studies have indicated that codelivery of ZFNs targeting the human *CCR5* and chemokine (C-X-C motif) receptor 4 (*CXCR4*) genes leads to protection from R5/X4 dual tropic HIV strains.^57,58^ To test whether multi-NLS ZFN protein delivery could mediate dual gene knockout, we generated ZFN proteins that targeted the *CXCR4*^58,59^ gene and contained one, three or four tandem NLS repeats. Three and four NLS repeats were chosen for this analysis since earlier studies revealed these proteins display the highest levels of cell permeability and genome editing activity. HeLa, K562, and Jurkat cells treated once with 4 μmol/l three- or four-NLS ZFN proteins showed a two- and threefold increase in *CXCR4* modification compared to one-NLS proteins, with Jurkat cells exhibiting mutagenesis frequencies up to 48% (**Figure 6a**). Flow cytometry analysis of CD4^+^ T cells treated with CXCR4 multi-NLS ZFN proteins also indicated high levels of knockdown of CXCR4 protein 5 days after treatment with 2 μmol/l three-NLS ZFN protein (**Supplementary Figure S6**). We treated CD4^+^ T cells simultaneously with 2 μmol/l three-NLS ZFN proteins targeting the *CCR5* and *CXCR4* genes. Analysis of DNA isolated from treated CD4^+^ cells indicated *CCR5* and *CXCR4* gene modification frequencies of >20 and >30%, respectively (**Figure 6b**), with no decrease in cleavage rates compared to individually targeted samples. Similar levels of mutagenesis were also observed 5 days after protein treatments, indicating the stability of these ZFN protein-induced modifications. Taken together, these findings indicate that coadministration of multi-NLS ZFN proteins facilitates high levels of modification of the *CCR5* and *CXCR4* genes in a therapeutically relevant setting.

## Discussion

Site-specific endonucleases, including homing endonucleases, ZFNs, TALENs, and CRISPR/Cas9, have dramatically expanded our ability to manipulate human cells and model organisms, and have the potential to treat the underlying genetic causes behind many diseases. While TALENs and CRISPR/Cas9 have emerged as especially facile genome engineering platforms due to the ease with which they can be customized and implemented, ZFNs remain potentially powerful tools for targeted gene therapy in humans.^47,51,52,57,60^ Indeed, a groundbreaking phase 1 clinical trial based on ZFN-mediated knockout of the *CCR5* gene indicated the safety and therapeutic efficacy of infusing modified autologous CD4^+^ T cells into patients with HIV.^15^ Despite this success, one major area of concern for many applications of nuclease-based genome engineering is safe and efficient delivery into relevant cell types. We previously reported that ZFN proteins possess the innate ability to cross cell membranes and mediate targeted gene knockout in human cells with reduced off-target effects.^27^ Unlike approaches that rely on expression from nucleic acids, nuclease protein delivery carries no risk of insertional mutagenesis, dramatically reduces the amount of time the nuclease is present within the cell, thereby reducing the frequency of off-target effects, and potentially overcomes many of the safety and regulatory hurdles associated with nuclease-based therapies by facilitating genome editing without introducing any genetic material into the cell. However, we previously found that high levels of gene knockout could only be achieved after consecutive ZFN protein treatments, a significant drawback that impacts both the scalability and scope of protein delivery for many *ex vivo* genome engineering processes.

Here, we show that genetic incorporation of tandem NLS repeats into the ZFN protein backbone enhances ZFN cell-permeability up to 13-fold and leads to highly efficient gene knockout in a broad range of human cell types, including CD4^+^ T cells, HSPCs and iPSCs, after only a single protein treatment. Multi-NLS ZFN proteins administrated directly into cells achieved rates of genomic modification that exceeded those achieved by plasmid DNA delivery via nucleofection, and rivaled those previously reported for many viral vector systems, including adenovirus.^47,57^ Despite the finding that five-NLS ZFN proteins nonspecifically cleaved plasmid DNA at higher rates than conventional (*i.e.*, one-NLS) ZFN proteins *in vitro*, all multi-NLS ZFN proteins tested displayed decreased off-target activity in K562 cells compared to ZFNs expressed from plasmid DNA, underlining the value of nuclease protein delivery for minimizing off-target effects. We suspect that the increased nonspecific cleavage activity observed with four- and five-NLS ZFN proteins compared to single-NLS domains could be attributed to excess binding energy between the highly positively charged NLS repeats and the negatively charged DNA backbone. For most cell types tested, four- and five-NLS ZFN proteins induced the highest levels of genomic modification despite the fact that *in vitro* cleavage analysis revealed these proteins display reduced activity compared to one-NLS ZFNs, indicating the increased genome editing rates stimulated by these proteins is largely due to their enhanced cell-penetrating activity. However, for precision genome engineering studies aimed at maximizing on- to off-target mutagenesis,^61^ we recommend using three-NLS ZFN proteins, which in many contexts displayed activity levels similar to four- and five-NLS proteins but induced fewer off-target effects.

We have also demonstrated the potential of multi-NLS ZFN protein delivery for inducing mutations into multiple genes within a bulk population of primary cells. Consecutive treatments with multi-NLS ZFN protein pairs targeting the genes for the HIV-1 coreceptors CCR5 and CXCR4 led to high levels of dual gene modification frequencies, indicating the potential of this approach for applications such as modification of autologous CD4^+^ T cells for treatment of HIV/AIDS. Multi-NLS ZFN proteins could also be used to enhance chimeric antigen receptor (CAR) therapies through *ex vivo* modification of the α or β chains of the T cell receptor in CAR^+^ T cells.^62,63^ In both cases, the genome modification rates afforded by multi-NLS ZFN proteins could improve the overall efficacy of each therapy. One obstacle that limited widespread adoption of ZFN protein delivery for such applications was the need to isolate and refold ZFN proteins from the *E. coli* insoluble fraction.^27^ Our current purification procedure is based entirely on purification from the soluble fraction and eliminates the need for time-consuming and laborious refolding procedures. Additional refinements of this procedure focused on eliminating proteolysis of the ZFN proteins, as well as biochemical analysis of the effects PTDs have on ZFN protein stability and expression, could further improve protein yield.

In summary, we show that incorporation of tandem NLS repeats enhances ZFN cell-permeability and that direct delivery of multi-NLS ZFN proteins to cells leads to high levels of genomic modification. This improvement to ZFN protein delivery has the potential to increase the safety and efficiency of *ex vivo* genome engineering processes and could be applicable to recently described zinc-finger-based protein delivery systems,^64,65^ cell-penetrating TALENs,^28^ Cas9 proteins^30,32^ and other genome-modifying enzymes.^66^

## Materials And Methods

*Plasmid construction.* The “left” and “right” one-NLS zinc-finger nuclease (ZFN) proteins designed to target the human *CCR5* gene were previously described.^47^ Both ZFN monomers were previously modified^27^ to contain the *Sharkey* cleavage domain.^48^ The ZFNs used in this study did not contain the obligate heterodimeric FokI architecture. ZFN genes were PCR-amplified from their respectively mammalian expression vectors (pVAX1-NH.CCR5.L/R; previously constructed in our laboratory)^27^ with the primers 5′ Two-NLS-ZF and 3′ Universal-ZF. All primer sequences are provided in **Supplementary Table S1**. PCR products were digested with XhoI and BamH1 and ligated into the same restriction sites of pVAX1-NH.CCR5.L/R or pET.CCR5.L.R/Sh (previously constructed in our laboratory) to generate the mammalian expression vectors pVAX-2NLS-CCR5.L/R, and the bacterial expression vectors pET.2NLS.CCR.L/R. Three-, four-, and five-NLS CCR5 ZFNs were constructed in an identical manner using the primers in **Supplementary Table S1**. One-, three-, and four-NLS ZFNs designed to target the human *CXCR4* gene^58^ were synthesized (GeneArt) and cloned into the same expression plasmids as described. Correct construction of each ZFN expression cassette was verified by sequence analysis (**Supplementary Table S2**).

*ZFN expression and purification.* Expression and purification methods were adapted from a previous study.^27^ pET-28 plasmids containing ZFNs were transformed into BL21 (DE3) cells. Overnight cultures grown from single colonies were inoculated into 700 ml lysogeny broth (LB) media supplemented with 50 μg/ml kanamycin, 90 μmol/l ZnCl_2_, 200 mmol/l NaCl and 0.2% glucose. Cultures were grown at 37 °C to an OD_600_ of 0.5, then moved to room temperature to grow until an OD_600_ of 0.8 was reached. Protein expression was induced with 0.1 mmol/l isopropyl-β-D-thiogalactopyranoside (IPTG) for 4 hours at room temperature. Cells from 700 ml cultures were pelleted by centrifugation at 5,000 rpm for 10 minutes and then resuspended in 20 ml ZFN binding buffer (20 mmol/l HEPES, pH 8.0, 2 mol/l NaCl, 1 mmol/l MgCl_2_, 90 μmol/l ZnCl_2_, and 10% glycerol). Following resuspension, 1 mmol/l β-mercaptoethanol, 1X complete inhibitor cocktail (Roche) and 0.1% Triton X-100 were added to the cells. Cells were then lysed by sonication and centrifuged at 25,000*g* for 30 minutes at 4 °C. The supernatant was cleared by its passing through a 0.45 μmol/l low protein-binding filter. Cleared cell lysate was incubated with 0.5 ml Ni-NTA agarose beads (QIAGEN) on a rotating table at 4 °C. The resin was then transferred to a column and washed with 10 ml Wash Buffer A (ZFN binding buffer + 5 mmol/l imidazole) followed by 5 ml Wash Buffer B (ZFN binding buffer + 35 mmol/l imidazole). ZFN proteins were eluted from the column with 4 ml Elution Buffer (ZFN binding buffer + 300 mmol/l imidazole). To each 0.5 ml fraction, 55 μl of 1 mol/l L-arginine (pH 7.2) was immediately added. Protein-containing fractions were identified by SDS-PAGE and combined. Purified ZFN proteins were buffer-exchanged to ZFN storage buffer (20 mmol/l HEPES, pH 8.0, 500 mmol/l NaCl, 1 mmol/l MgCl_2_, 90 μmol/l ZnCl_2_, 10% glycerol, and 100 mmol/l L-Arg) and concentrated to at least 20 μmol/l using 10,000 Dalton Molecular Weight Cut-Off Vivaspin Spin Concentrators (VivaProducts). Concentrated ZFN proteins were filter-sterilized using a 0.2 μm syringe filter and stored at −80 °C for cell culture application.

*In vitro cleavage assay. In vitro* DNA cleavage was performed as described^27,48^ in a volume of 10 μl containing 100 ng of substrate DNA and varying concentrations of ZFN proteins in cleavage buffer (10 mmol/l Tris–HCl, pH 7.9, 50 mmol/l NaCl, 10 mmol/l MgCl_2_, 1 mmol/l DTT, 90 μmol/l ZnCl_2_, and 100 mmol/l L-Arg). The reaction mixture was incubated at room temperature for 1 hour and quenched with 1X agarose gel loading buffer. The product was visualized on a 1% agarose gel.

*Cell culture.* The HEK293-based EGFP reporter cell line used in this study was constructed and analyzed by flow cytometry as previously described.^27,48^ HeLa, K562, and Jurkat cells were obtained from American Type Culture Collection (ATCC). HEK293, HeLa and reporter cells were maintained in Dulbecco's modified Eagle's medium (DMEM) supplemented with 10% (vol/vol) fetal bovine serum (FBS), 100 Units (U) /ml penicillin and 100 U/ml streptomycin. K562 and Jurkat cells were maintained in RPMI 1640 medium containing 10% (vol/vol) FBS, 100 U/ml penicillin and 100 U/ml streptomycin. All cells were cultured at 37 °C in a humidified atmosphere with 5% CO_2_. For transfections, reporter cells were seeded onto 24-well plates at a density of 2 × 10^5^ cells per well. At 24 hours after seeding, reporter cells were transfected with 100 ng of “left” and “right” pVAX1 one-NLS ZFN expression vector using Lipofectamine 2000 (Invitrogen) according to the manufacturer's instructions. For nucleofections, 2 × 10^5^ K562 cells were seeded onto 24-well plates at a density of 2 × 10^5^ cells per well. At 24 hours after seeding, cells were nucleofected with 1 μg of “left” and “right” pVAX1 one-NLS ZFN expression vectors using a 4D-Nucleofector System (Lonza) according to the manufacturer's instructions.

Peripheral blood mononuclear cells (PBMCs) were isolated from normal human donors through The Scripps Research Institute Normal Blood Donor Program. CD4^+^ cells were purified from PBMCs by negative selection using the EasySep Human CD4^+^ T cell Enrichment Kit (StemCell Technologies). To activate and expand the isolated CD4^+^ cells, 1 × 10^6^ cells/well were seeded in a 24-well plate and incubated in RPMI 1640 medium containing 10% FBS, 100 U/ml penicillin, 100 U/ml streptomycin, 25 μl/ml CD3/CD28 T-activator Dynabeads (Dynal/Life Technologies), and 50 U/ml recombinant interleukin-2 (rIL-2; R&D Systems). Bone marrow-derived CD34^+^ hematopoietic stem/progenitor cells (HSPCs) were obtained from AllCells, LLC at a purity of >97% (**Supplementary Figure S7**). Cells were maintained in StemSpan serum-free expansion medium (StemCell Technologies) supplemented with 50 ng/ml rhSCF/c-Kit, 20 ng/ml rhIL-3, and 20 ng/ml rhIL-6 (R&D Systems). Induced pluripotent stem cells (iPSCs) were previously generated from keratinocytes donated by a healthy anonymous individual (KiPSC68)^67^ and maintained in feeder-free conditions with mTresR1 (Stem Cell Technologies) on Matrigel-coated surfaces (Fisher). Cells were passaged every 5–7 d by Gentle Cell Dissociation Reagent (Stem Cell Technologies).

*Protein treatments.* HEK293, HeLa, K562, and Jurkat cells were seeded in a 24-well plate at 2 × 10^5^ cells/well. Low-passage iPSCs were dissociated by Accutase (Stem Cell Technologies) and seeded at a density of 2–3 × 10^4^ cells/cm^2^ in the presence of ROCK inhibitor Y27632 for 1 d to facilitate attachment and survival. At 24 hours after seeding, growth medium was removed and cells were rinsed with DMEM serum-free medium (SFM). ZFN proteins were prepared at various concentrations in 250 μl SFM containing 90 μmol/l ZnCl_2_ and 100 mmol/l L-Arg, and incubated with cells at 37 °C for 1 hour in a humidified atmosphere with 5% CO_2_. ZFN-containing medium was then replaced with full growth medium, and transduced cells were transiently cold shocked^68^ at 30 °C for 24 hours. Cells were then moved to 37 °C for 24 hours and harvested for further analysis. Triplicate experiments for both CD4^+^ cells and HSPCs were performed in 96-well plates seeded with 5 × 10^4^ cells/well. Treatments were performed with equimolar amounts of “left” and “right” ZFN proteins' with identical NLS repeat lengths. To measure CXCR4 expression by flow cytometry, 48 hours after treatment, CD4^+^ T cells were harvested, washed with PBS, and incubated on ice for 60 minutes with phycoerythrin (PE)-conjugated anti-CXCR4 antibody (Clone 12G5; BD Biosciences). Cells were washed and resuspended with PBS/1% FBS, and cell-surface expression of CXCR4 was measured by flow cytometry (FACScan Dual Laser Cytometer; BD Biosciences; FACSDiva software). For each sample, 10,000 live events were collected, and data was analyzed using FlowJo (Tree Star).

*Internalization assay.* ZFN proteins were buffer-exchanged to conjugation solution (20 mmol/l HEPES, pH 7.2, 500 mmol/l NaCl, 1 mmol/l MgCl_2_, 90 μmol/l ZnCl_2_, and 100 mmol/l L-Arg) and subsequently incubated with tenfold molar excess fluorescein-5-maleimide (Pierce) at 4 °C overnight. Zeba Spin Desalting Columns (Pierce) were used to remove unreacted dye. The concentration of labeled ZFN protein was determined by SDS-PAGE using BSA standards. For protein treatments, HEK293 cells were seeded onto a 24-well plate at a density of 2 × 10^5^ cells/well. At 24 hours after seeding, cells were incubated with 1 μmol/l fluorescein-conjugated ZFN proteins at 37 °C for 1 hour. After treatment, cells were washed three times with PBS containing 0.5 mg/ml heparin and harvested for analysis. ZFN protein treated cells were resuspended in PBS/1% FBS and fluorescence was measured by flow cytometry using the FITC channel (FACScan Dual Laser Cytometer; BD Biosciences; FACSDiva software). For each sample, 10,000 live events were collected, and data was analyzed using FlowJo (Tree Star).

*Surveyor nuclease assay.* Genomic DNA was isolated from transduced and transfected cells using QuickExtract DNA Extraction Solution (Epicentre), and the frequency of endogenous gene disruption was evaluated using the Surveyor nuclease assay (Transgenomics) as previously described.^49^ Cleavage products were visualized by PAGE and the frequency of gene disruption was determined by measuring the ratio of cleaved to uncleaved substrate, as described.^49^ The *CCR5* and *CXCR4* genes were amplified from the genomic DNA using the Expand High Fidelity *Taq* System (Roche) using the primers provided in **Supplementary Table S1** and cloned into the plasmid pUC19. Sequence analysis was performed on individual cloned transformants as described.^27^

*Cell viability.* Stimulated CD4^+^ T cells were seeded onto 96-well plates at 5 × 10^4^ cells/well in RPMI1640 medium containing 10% FBS, 100 U/ml penicillin, 100 U/ml streptomycin, and 50 U/ml rIL-2. At 24 hours after seeding, cells were treated once with ZFN proteins as described above. Cell viability was measured 2 days after ZFN protein treatments using the Cell Proliferation Kit II (XTT; Roche Applied Science), according to the manufacturer's instructions.

**SUPPLEMENTARY MATERIAL** **Figure S1.** Purification of ZFN proteins fused to the protein transduction domains penetratin and transportan. **Figure S2.** The protein transduction domains penetratin and transportan do not enhance ZFN protein activity. **Figure S3.** SDS-PAGE and *in vitro* cleavage analysis of multi-NLS ZFN proteins. **Figure S4.** Genomic modifications induced by transiently expressed multi-NLS ZFNs. **Figure S5.** Sequence analysis of modified *CCR5* alleles from stimulated human CD4^+^ T cells, hematopoietic stem/progenitor cells and induced pluripotent stem cells. **Figure S6.** CXCR4 expression in CD4^+^ T cells treated with three-NLS CXCR4 ZFN proteins. **Figure S7.** Purity of CD34^+^ hematopoietic stem/progenitor cells (HSPCs). **Table S1.** Primer sequences used in this study. **Table S2.** Amino acid sequences of the ZFN proteins used in this study.
