# Supplementary material for: Improved Cell-Penetrating Zinc-Finger Nuclease Proteins for Precision Genome Engineering
Source: Mol Ther Nucleic Acids. 2015 Mar 10;4(3):e232–. doi: 10.1038/mtna.2015.6 (PMC4354341; doi:10.1038/mtna.2015.6)
Supplement: Supplementary Information [file mtna20156x1.doc]

**Supplementary Information:**

**Improved cell-penetrating zinc-finger nuclease proteins for precision genome engineering**

Jia Liu^1, 2, 3, 4, 5^, Thomas Gaj^1, 2, 3, 5^, Mark C. Wallen^1, 2, 3^ and Carlos F. Barbas III^1, 2, 3, 6^

^1^The Skaggs Institute for Chemical Biology, The Scripps Research Institute, La Jolla, CA, USA; ^2^Department of Chemistry, The Scripps Research Institute, La Jolla, CA, USA; ^3^Department of Cell and Molecular Biology, The Scripps Research Institute, La Jolla, CA, USA; ^4^Shanghai Institute for Advanced Immunochemical Studies (SIAIS), ShanghaiTech University, Shanghai, China.

^5^The authors contributed equally to this work

^6^Deceased.

Correspondence:

Jia Liu, Shanghai Institute for Advanced Immunochemical Studies (SIAIS), ShanghaiTech University, Shanghai, China. Email: email: liujia@shanghaitech.edu.cn

Thomas Gaj, Department of Chemical and Biomolecular Engineering, University of California, Berkeley, Berkeley, CA, USA. Email: gaj@berkeley.edu

Short title: Improved cell-penetrating ZFN proteins

Keywords: genome editing / zinc-finger nuclease / protein delivery /

**SUPPLEMENTARY FIGURES**

**
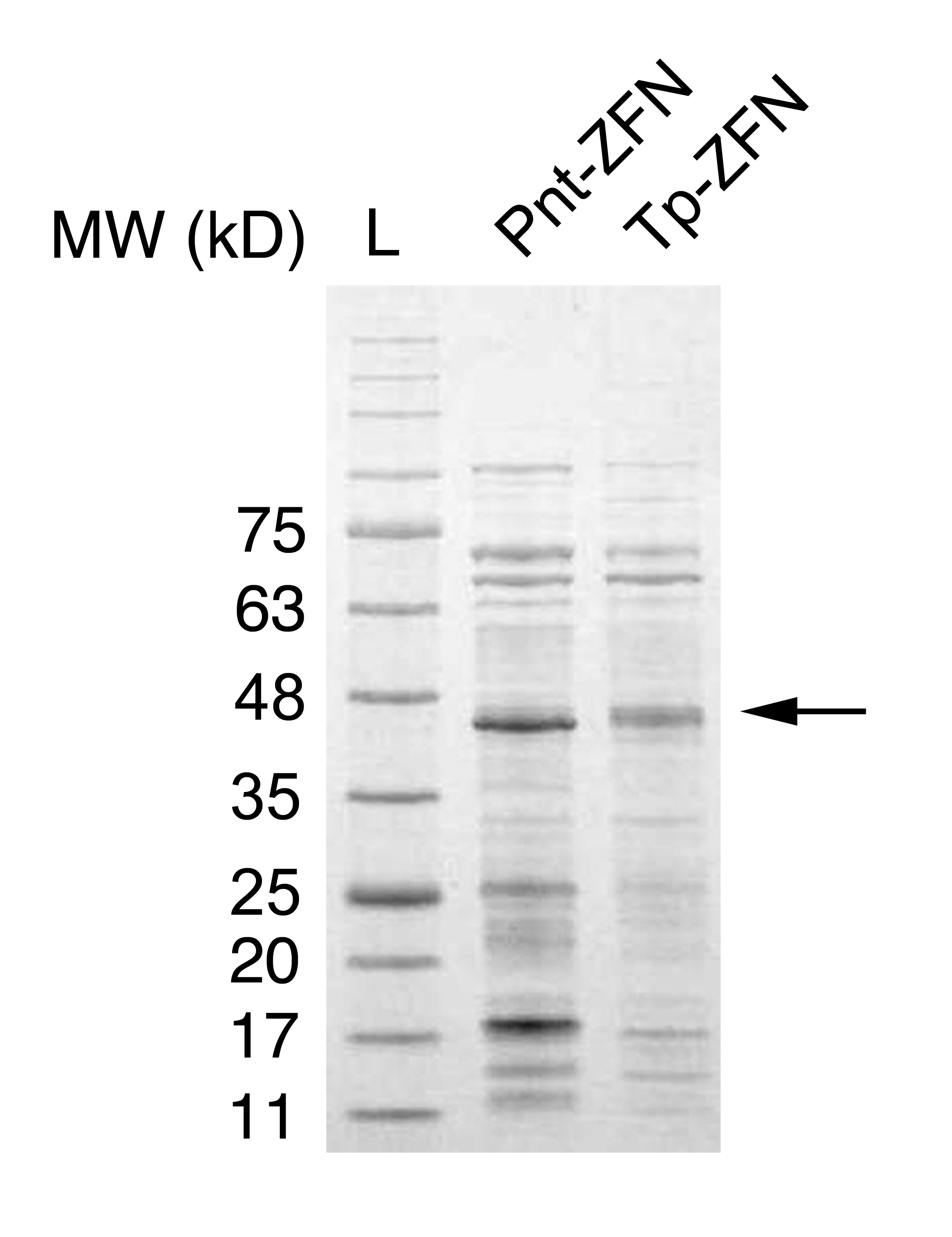
**

**Figure S1. Purification of ZFN proteins fused to the protein transduction domains penetratin and transportan.** Coomassie blue stained SDS-PAGE of ‘right’ CCR5 ZFN proteins fused to penetratin (Pnt) and transportan (Tp) purified from the soluble fraction of *E. coli* lysate. Molecular weight (MW) standards indicated. Arrow indicates the anticipated MW of ZFN fusion proteins

**
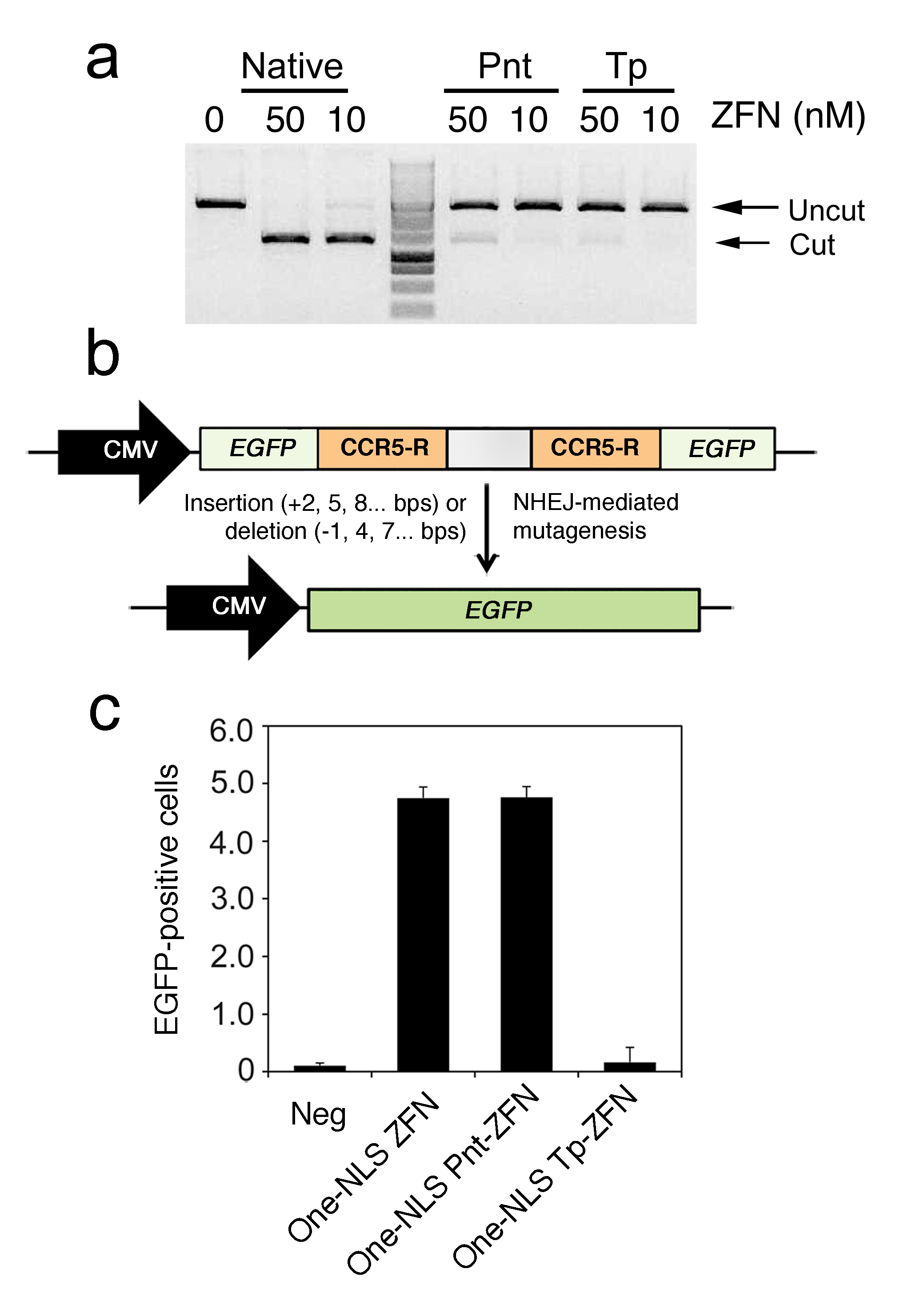
**

**Figure S2. The protein transduction domains penetratin and transportan do not enhance ZFN protein activity. (a)** *In vitro* cleavage assay of ‘right’ penetratin (Pnt) and transportan (Tp) CCR5 ZFN fusion proteins incubated with 100 ng substrate DNA. Cut and uncut substrate DNA indicated. Native denotes one-NLS ZFN protein. **(b)** Schematic representation of the HEK293 EGFP reporter system used to evaluate PTD-ZFN protein activity. The expression of an integrated EGFP gene was disabled by a frame-shift mutation introduced by a symmetrical ZFN cleavage site. One-third of all ZFN-induced DSBs restore the EGFP reading frame. **“**CCR5-R” indicates the “right” CCR5 ZFN protein binding sites. Note, the EGFP reporter system shown here is identical to the one depicted in Figure 1B **(c)** Percentage of EGFP-positive cells measured by flow cytometry following one treatment with 1 μM ‘right’ CCR5 ZFN fusion proteins. Each fusion protein contained one-NLS domain. “One-NLS ZFN” indicates native protein. “Neg” indicates cells treated with serum-free medium. Bars represent ± s.d. (n = 3).

**
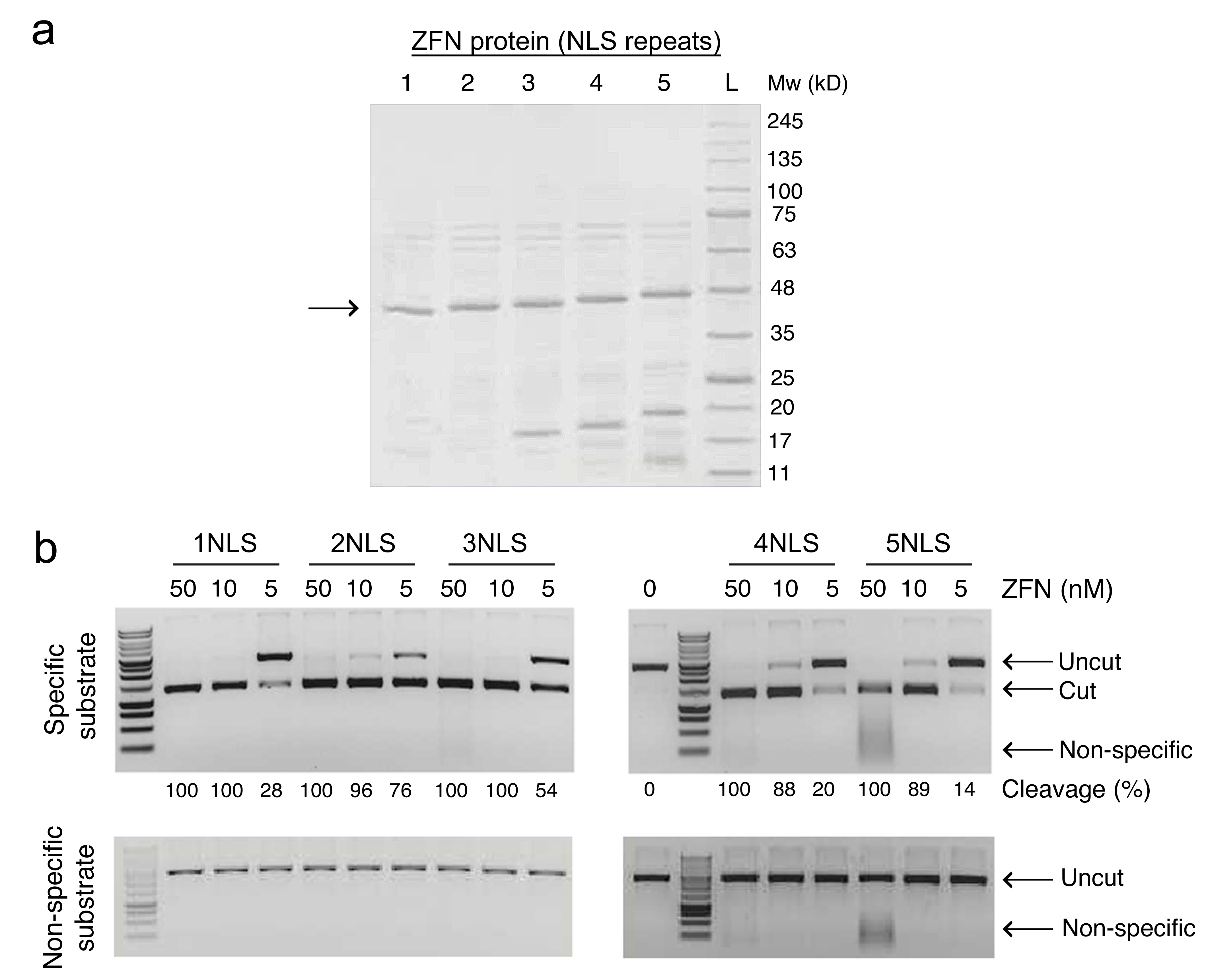
**

**Figure S3. SDS-PAGE and *in vitro* cleavage analysis of multi-NLS ZFN proteins. (a)** Coomassie blue stained SDS-PAGE of one-, two-, three-, four- and five-NLS ‘right’ CCR5 ZFN proteins purified from the soluble fraction of *E. coli* lysate. Protein molecular weight (MW) standards indicated. Arrow indicates the anticipated MW of one-NLS ZFN protein. (**b)** *In vitro* cleavage assay of 50, 10 or 5 nM one-, two-, three-, four- and five-NLS ‘right’ CCR5 ZFN proteins with 100 ng of **(top)** substrate or **(bottom)** non-substrate DNA. **(Top)** Cut and uncut substrate DNA indicated. **(Bottom)** Uncut and non-specifically cut non-substrate DNA indicated.

**
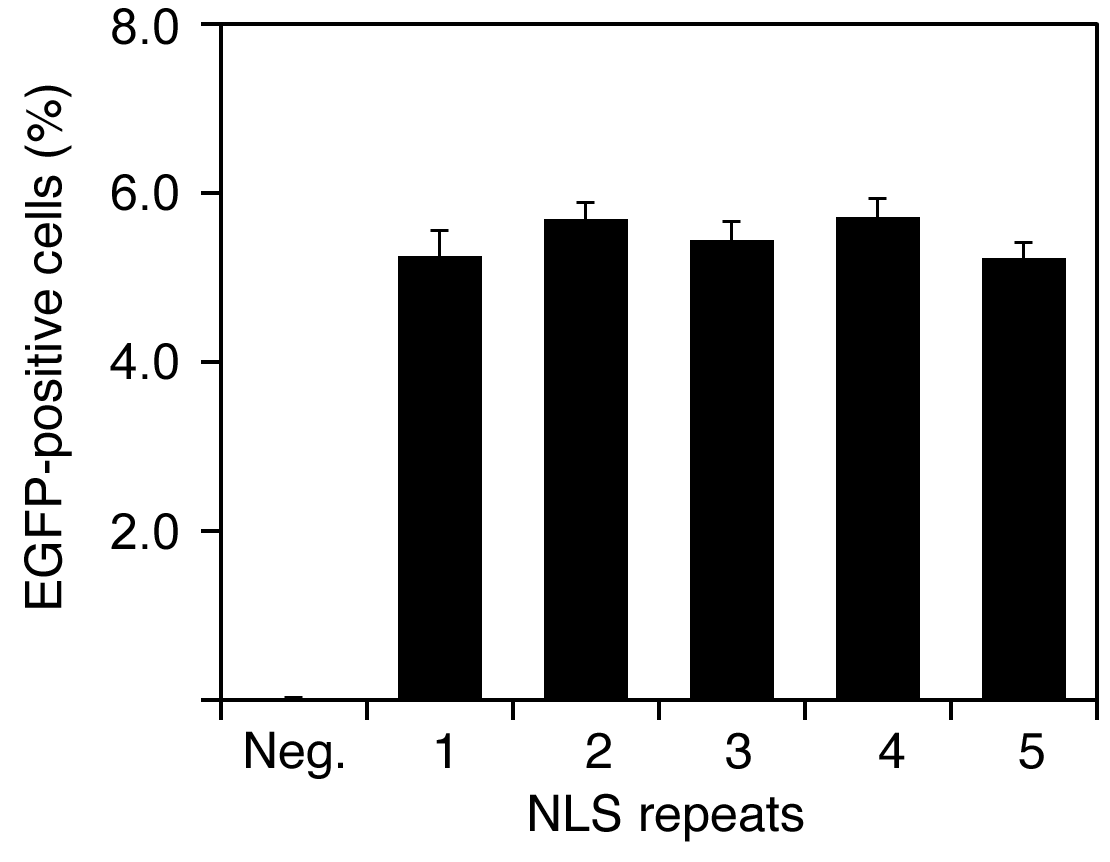
**

**Figure S4. Genomic modifications induced by transiently expressed multi-NLS ZFNs.** Percentage of EGFP-positive reporter cells measured by flow cytometry following Lipofectamine-mediated transfection of 100 ng one-, two-, three-, four- and five-NLS CCR5 ‘right’ ZFN expression vector. EGFP-positive cells were measured 72 h after transfection. “Neg.” indicates reporter cells transfected with empty vector. Bars represent ± s.d. (n = 3).


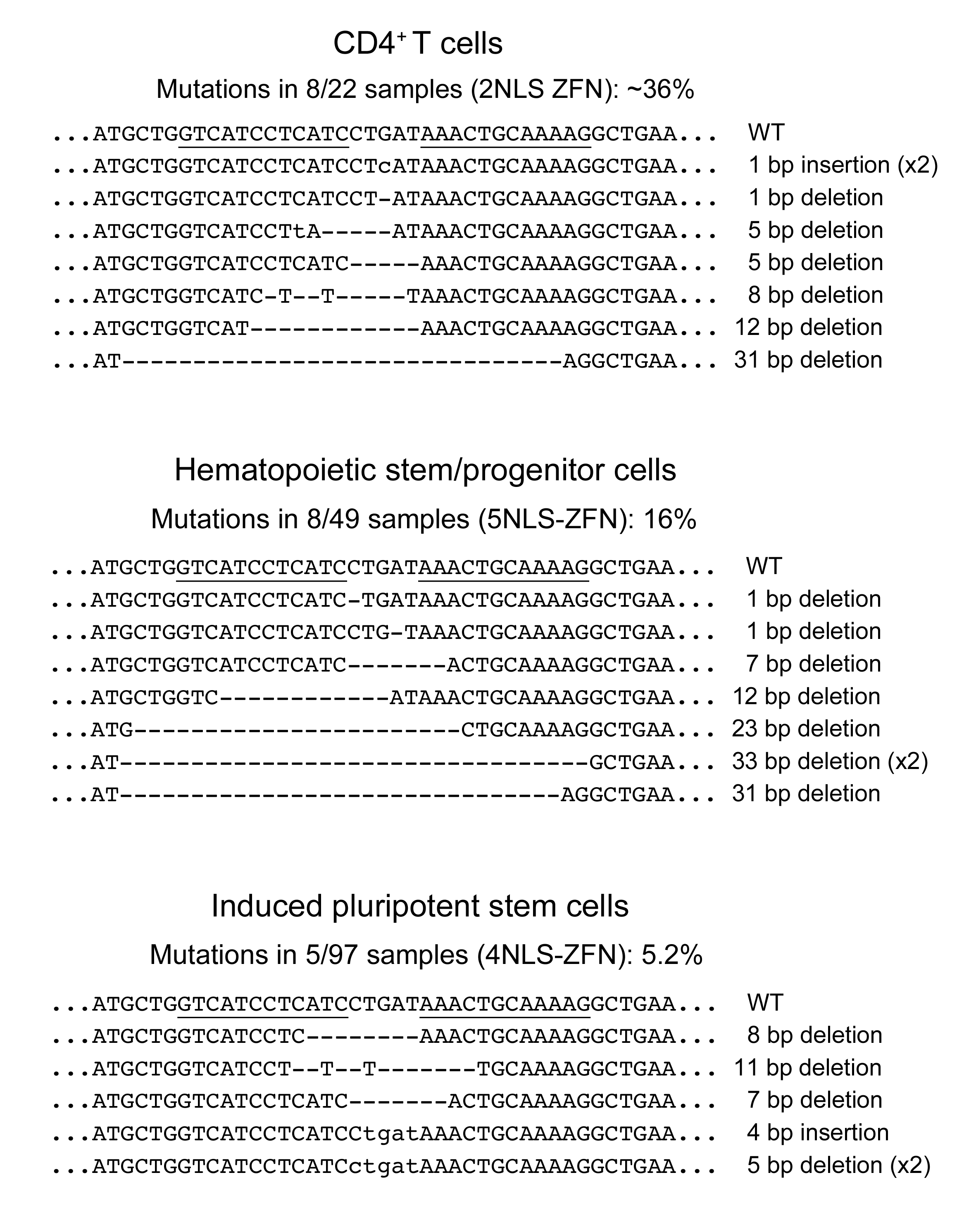


**Figure S5. Sequence analysis of modified *CCR5* alleles from stimulated human CD4^+^ T cells, hematopoietic stem/progenitor cells and induced pluripotent stem cells.** ZFN protein binding sites are underlined. Number of sequences with ZFN-induced insertions and deletions are indicated.

**
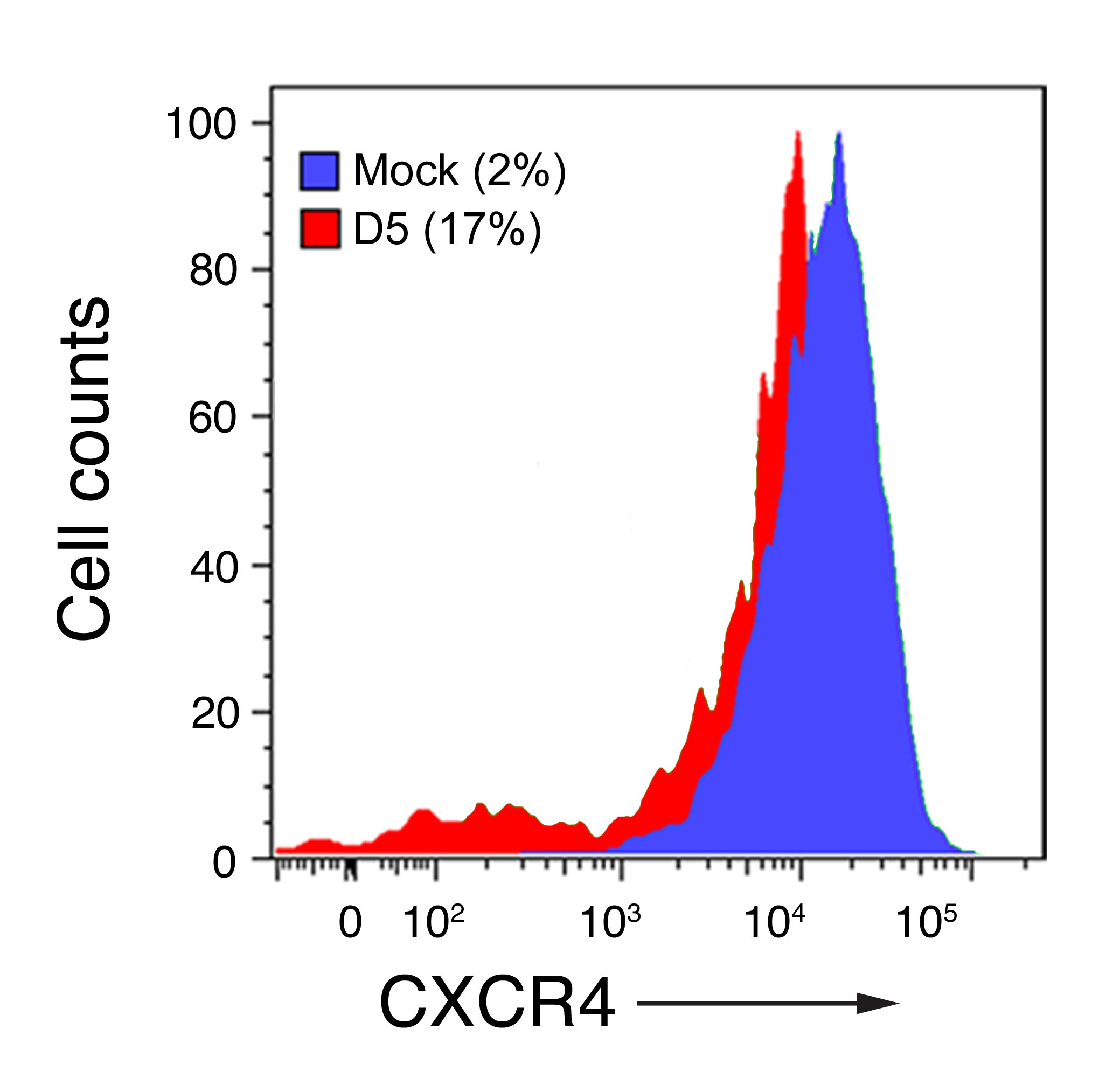
**

**Figure S6. CXCR4 expression in CD4^+^ T cells treated with three-NLS CXCR4 ZFN proteins.** Mean percentage of CXCR4 negative CD4^+^ cells measured by flow cytometry after one treatment with 2 μM three-NLS CXCR4 ZFN proteins. CXCR4 levels were measured 5 d after protein treatment. Mock indicates cells treated with serum-free medium.


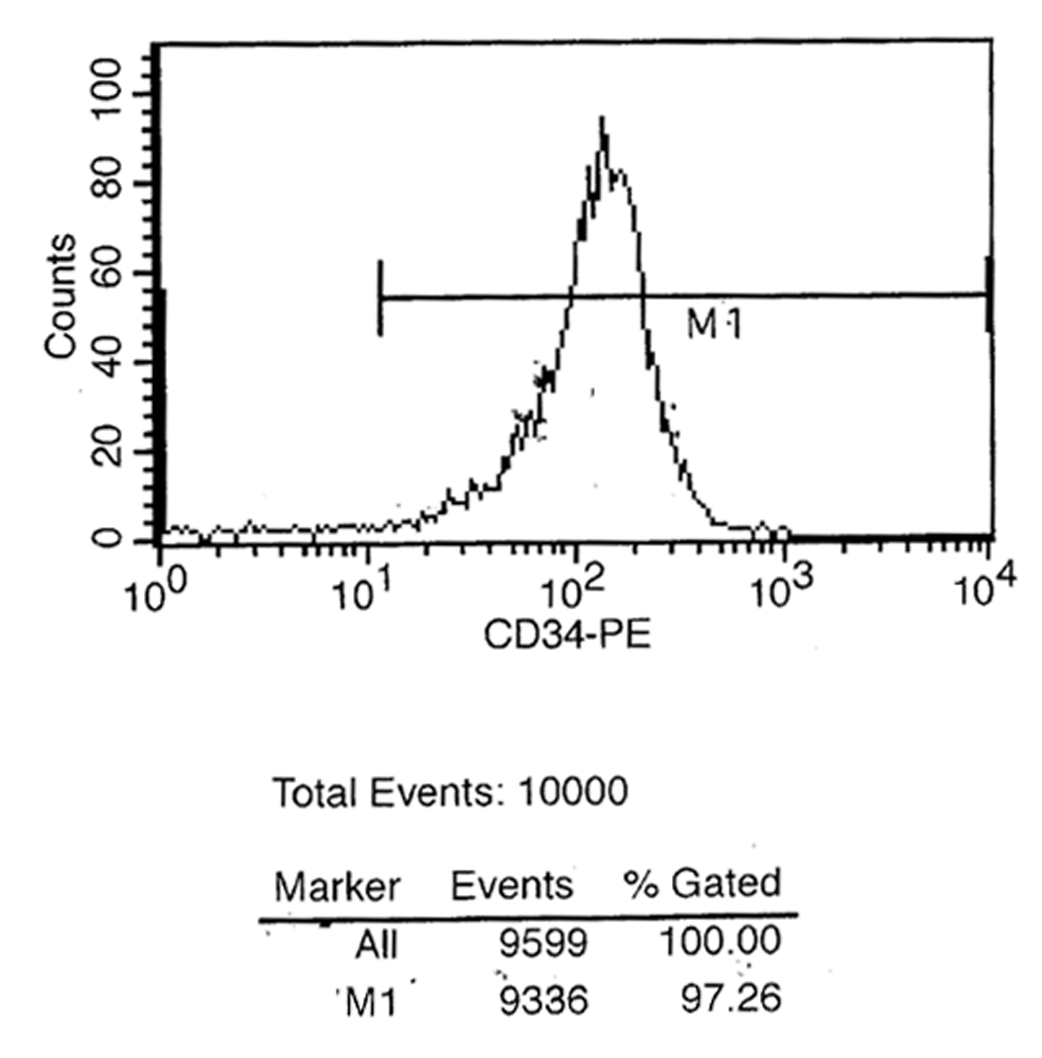


**Figure S7. Purity of CD34^+^ hematopoietic stem/progenitor cells (HSPCs).** Flow cytometry analysis of CD34^+^ HSPCs obtained from AllCells, LLC. Purity of cells estimated to be 97%. Analysis performed and provided by AllCells, LLC.

**SUPPLEMENTARY TABLES**

| Primer name | Sequence |
| --- | --- |
| Two-NLS-ZF | GGTCTCGAGCCCGGGATGGCCCCCAAGAAAAAGCGGAAAGTGGGCATCCACGGCGTGCCTGCCGCCATGGCCGAGCGGCCCTTC |
| Three-NLS-ZF | CCGCTCGAGCCAAAGAAGAAACGGAAAGTACCCGGGATGGCCCCCAAG |
| Four-NLS-ZF | CCGCTCGAGCCAAAGAAGAAACGGAAAGTAGGCGGCTCCCCCAAAAAGAAGCGAAAAGTGCCCGGGATGGCCCCCAAG |
| Five-NLS-ZF | CCGCTCGAGCCAAAGAAGAAACGGAAAGTAGGCGGCTCCCCCAAAAAGAAGCGAAAAGTGGGGGGGTCCCCCAAGAAGAAGCGGAAGGTACCCGGGATGGCCCCCAAG |
| Universal-ZF | TTTGACTAGTTGGGATCCCCGCAG |
| 5’ CCR5 External | GCTTGAGCCCAGGAGTTCGA |
| 3’ CCR5 External | AACTGAGCTTGCTCGCTCGG |
| 5’ CCR5 Internal (BamHI) | CGCGGATCCACAGTTTGCATTCATGGAGGGC |
| 3’ CCR5 Internal (EcoRI) | CCGGAATTCACCGTCCTGGCTTTTAAAGC |
| 5’ CXCR4 (XbaI) | CGCTCTAGACAGTCAACCTCTACAGCAGTGTCC |
| 3’ CXCR4 (EcoRI) | CCGGAATTCGGAGTGTGACAGCTTGGAGATG |

**Table S1.** Primer sequences used in this study. Restriction sites are underlined.

**>One-NLS CCR5 ZFN Left**

MGSSHHHHHHSSGLVPRGSHMPKKKRKVLEAAMAERPFQCRICMRNFSDRSNLSRHIRTHTGEKPFACDICGRKFAISSNLNSHTKIHTGSQKPFQCRICMRNFSRSDNLARHIRTHTGEKPFACDICGRKFATSGNLTRHTKIHLRGSQLVKSELEEKKSELRHKLKYVPHEYIELIEIARNPTQDRILEMKVMEFFMKVYGYRGEHLGGSRKPDGAIYTVGSPIDYGVIVDTKAYSGGYNLPIGQADEMQRYVEENQTRNKHINPNEWWKVYPSSVTEFKFLFVSGHFKGNYKAQLTRLNHITNCNGAVLSVEELLIGGEMIKAGTLTLEEVRRKFNNGEINF

**>Two-NLS CCR5 ZFN Left**

MGSSHHHHHHSSGLVPRGSHMPKKKRKVLEPGMAPKKKRKVGIHGVPAAMAERPFQCRICMRNFSDRSNLSRHIRTHTGEKPFACDICGRKFAISSNLNSHTKIHTGSQKPFQCRICMRNFSRSDNLARHIRTHTGEKPFACDICGRKFATSGNLTRHTKIHLRGSQLVKSELEEKKSELRHKLKYVPHEYIELIEIARNPTQDRILEMKVMEFFMKVYGYRGEHLGGSRKPDGAIYTVGSPIDYGVIVDTKAYSGGYNLPIGQADEMQRYVEENQTRNKHINPNEWWKVYPSSVTEFKFLFVSGHFKGNYKAQLTRLNHITNCNGAVLSVEELLIGGEMIKAGTLTLEEVRRKFNNGEINF

**>Three-NLS CCR5 ZFN Left**

MGSSHHHHHHSSGLVPRGSHMPKKKRKVLEPKKKRKVPGMAPKKKRKVGIHGVPAAMAERPFQCRICMRNFSDRSNLSRHIRTHTGEKPFACDICGRKFAISSNLNSHTKIHTGSQKPFQCRICMRNFSRSDNLARHIRTHTGEKPFACDICGRKFATSGNLTRHTKIHLRGSQLVKSELEEKKSELRHKLKYVPHEYIELIEIARNPTQDRILEMKVMEFFMKVYGYRGEHLGGSRKPDGAIYTVGSPIDYGVIVDTKAYSGGYNLPIGQADEMQRYVEENQTRNKHINPNEWWKVYPSSVTEFKFLFVSGHFKGNYKAQLTRLNHITNCNGAVLSVEELLIGGEMIKAGTLTLEEVRRKFNNGEINF

**>Four-NLS CCR5 ZFN Left**

MGSSHHHHHHSSGLVPRGSHMPKKKRKVLEPKKKRKVGGSPKKKRKVPGMAPKKKRKVGIHGVPAAMAERPFQCRICMRNFSDRSNLSRHIRTHTGEKPFACDICGRKFAISSNLNSHTKIHTGSQKPFQCRICMRNFSRSDNLARHIRTHTGEKPFACDICGRKFATSGNLTRHTKIHLRGSQLVKSELEEKKSELRHKLKYVPHEYIELIEIARNPTQDRILEMKVMEFFMKVYGYRGEHLGGSRKPDGAIYTVGSPIDYGVIVDTKAYSGGYNLPIGQADEMQRYVEENQTRNKHINPNEWWKVYPSSVTEFKFLFVSGHFKGNYKAQLTRLNHITNCNGAVLSVEELLIGGEMIKAGTLTLEEVRRKFNNGEINF

**>Five-NLS CCR5 ZFN Left**

MGSSHHHHHHSSGLVPRGSHMPKKKRKVLEPKKKRKVGGSPKKKRKVGGSPKKKRKVPGMAPKKKRKVGIHGVPAAMAERPFQCRICMRNFSDRSNLSRHIRTHTGEKPFACDICGRKFAISSNLNSHTKIHTGSQKPFQCRICMRNFSRSDNLARHIRTHTGEKPFACDICGRKFATSGNLTRHTKIHLRGSQLVKSELEEKKSELRHKLKYVPHEYIELIEIARNPTQDRILEMKVMEFFMKVYGYRGEHLGGSRKPDGAIYTVGSPIDYGVIVDTKAYSGGYNLPIGQADEMQRYVEENQTRNKHINPNEWWKVYPSSVTEFKFLFVSGHFKGNYKAQLTRLNHITNCNGAVLSVEELLIGGEMIKAGTLTLEEVRRKFNNGEINF

**>One-NLS CCR5 ZFN Right**

MGSSHHHHHHSSGLVPRGSHMPKKKRKVLEAAMAERPFQCRICMRNFSRSDNLSVHIRTHTGEKPFACDICGRKFAQKINLQVHTKIHTGEKPFQCRICMRNFSRSDVLSEHIRTHTGEKPFACDICGRKFAQRNHRTTHTKIHLRGSQLVKSELEEKKSELRHKLKYVPHEYIELIEIARNPTQDRILEMKVMEFFMKVYGYRGEHLGGSRKPDGAIYTVGSPIDYGVIVDTKAYSGGYNLPIGQADEMQRYVEENQTRNKHINPNEWWKVYPSSVTEFKFLFVSGHFKGNYKAQLTRLNHITNCNGAVLSVEELLIGGEMIKAGTLTLEEVRRKFNNGEINF

**>Two-NLS CCR5 ZFN Right**

MGSSHHHHHHSSGLVPRGSHMPKKKRKVLEPGMAPKKKRKVGIHGVPAAMAERPFQCRICMRNFSDRSNLSRHIRTHTGEKPFACDICGRKFAISSNLNSHTKIHTGSQKPFQCRICMRNFSRSDNLARHIRTHTGEKPFACDICGRKFATSGNLTRHTKIHLRGSQLVKSELEEKKSELRHKLKYVPHEYIELIEIARNPTQDRILEMKVMEFFMKVYGYRGEHLGGSRKPDGAIYTVGSPIDYGVIVDTKAYSGGYNLPIGQADEMQRYVEENQTRNKHINPNEWWKVYPSSVTEFKFLFVSGHFKGNYKAQLTRLNHITNCNGAVLSVEELLIGGEMIKAGTLTLEEVRRKFNNGEINF

**>Three-NLS CCR5 ZFN Right**

MGSSHHHHHHSSGLVPRGSHMPKKKRKVLEPKKKRKVPGMAPKKKRKVGIHGVPAAMAERPFQCRICMRNFSRSDNLSVHIRTHTGEKPFACDICGRKFAQKINLQVHTKIHTGEKPFQCRICMRNFSRSDVLSEHIRTHTGEKPFACDICGRKFAQRNHRTTHTKIHLRGSQLVKSELEEKKSELRHKLKYVPHEYIELIEIARNPTQDRILEMKVMEFFMKVYGYRGEHLGGSRKPDGAIYTVGSPIDYGVIVDTKAYSGGYNLPIGQADEMQRYVEENQTRNKHINPNEWWKVYPSSVTEFKFLFVSGHFKGNYKAQLTRLNHITNCNGAVLSVEELLIGGEMIKAGTLTLEEVRRKFNNGEINF

**>Four-NLS CCR5 ZFN Right**

MGSSHHHHHHSSGLVPRGSHMPKKKRKVLEPKKKRKVGGSPKKKRKVPGMAPKKKRKVGIHGVPAAMAERPFQCRICMRNFSRSDNLSVHIRTHTGEKPFACDICGRKFAQKINLQVHTKIHTGEKPFQCRICMRNFSRSDVLSEHIRTHTGEKPFACDICGRKFAQRNHRTTHTKIHLRGSQLVKSELEEKKSELRHKLKYVPHEYIELIEIARNPTQDRILEMKVMEFFMKVYGYRGEHLGGSRKPDGAIYTVGSPIDYGVIVDTKAYSGGYNLPIGQADEMQRYVEENQTRNKHINPNEWWKVYPSSVTEFKFLFVSGHFKGNYKAQLTRLNHITNCNGAVLSVEELLIGGEMIKAGTLTLEEVRRKFNNGEINF

**>Five-NLS CCR5 ZFN Right**

MGSSHHHHHHSSGLVPRGSHMPKKKRKVLEPKKKRKVGGSPKKKRKVGGSPKKKRKVPGMAPKKKRKVGIHGVPAAMAERPFQCRICMRNFSRSDNLSVHIRTHTGEKPFACDICGRKFAQKINLQVHTKIHTGEKPFQCRICMRNFSRSDVLSEHIRTHTGEKPFACDICGRKFAQRNHRTTHTKIHLRGSQLVKSELEEKKSELRHKLKYVPHEYIELIEIARNPTQDRILEMKVMEFFMKVYGYRGEHLGGSRKPDGAIYTVGSPIDYGVIVDTKAYSGGYNLPIGQADEMQRYVEENQTRNKHINPNEWWKVYPSSVTEFKFLFVSGHFKGNYKAQLTRLNHITNCNGAVLSVEELLIGGEMIKAGTLTLEEVRRKFNNGEINF

**>One-NLS CXCR4 ZFN Left**

MGSSHHHHHHSSGLVPRGSHMPKKKRKVLEAAMAERPFQCRICMRNFSDRSALSRHIRTHTGEKPFACDICGRKFARSDDLTRHTKIHTGSQKPFQCRICMRNFSQSGNLARHIRTHTGEKPFACDICGRKFAQSGSLTRHTKIHLRGSQLVKSELEEKKSELRHKLKYVPHEYIELIEIARNPTQDRILEMKVMEFFMKVYGYRGEHLGGSRKPDGAIYTVGSPIDYGVIVDTKAYSGGYNLPIGQADEMQRYVEENQTRNKHINPNEWWKVYPSSVTEFKFLFVSGHFKGNYKAQLTRLNHITNCNGAVLSVEELLIGGEMIKAGTLTLEEVRRKFNNGEINF

**>Three-NLS CXCR4 ZFN Left**

MGSSHHHHHHSSGLVPRGSHMPKKKRKVLEPKKKRKVPGMAPKKKRKVGIHGVPAAMAERPFQCRICMRNFSDRSALSRHIRTHTGEKPFACDICGRKFARSDDLTRHTKIHTGSQKPFQCRICMRNFSQSGNLARHIRTHTGEKPFACDICGRKFAQSGSLTRHTKIHLRGSQLVKSELEEKKSELRHKLKYVPHEYIELIEIARNPTQDRILEMKVMEFFMKVYGYRGEHLGGSRKPDGAIYTVGSPIDYGVIVDTKAYSGGYNLPIGQADEMQRYVEENQTRNKHINPNEWWKVYPSSVTEFKFLFVSGHFKGNYKAQLTRLNHITNCNGAVLSVEELLIGGEMIKAGTLTLEEVRRKFNNGEINF

**>Four-NLS CXCR4 ZFN Left**

MGSSHHHHHHSSGLVPRGSHMPKKKRKVLEPKKKRKVGGSPKKKRKVPGMAPKKKRKVGIHGVPAAMAERPFQCRICMRNFSDRSALSRHIRTHTGEKPFACDICGRKFARSDDLTRHTKIHTGSQKPFQCRICMRNFSQSGNLARHIRTHTGEKPFACDICGRKFAQSGSLTRHTKIHLRGSQLVKSELEEKKSELRHKLKYVPHEYIELIEIARNPTQDRILEMKVMEFFMKVYGYRGEHLGGSRKPDGAIYTVGSPIDYGVIVDTKAYSGGYNLPIGQADEMQRYVEENQTRNKHINPNEWWKVYPSSVTEFKFLFVSGHFKGNYKAQLTRLNHITNCNGAVLSVEELLIGGEMIKAGTLTLEEVRRKFNNGEINF

**>One-NLS CXCR4 ZFN Right**

MGSSHHHHHHSSGLVPRGSHMPKKKRKVLEAAMAERPFQCRICMRNFSRSDSLLRHIRTHTGEKPFACDICGRKFARSDHLTTHTKIHTGSQKPFQCRICMRNFSRSDSLSAHIRTHTGEKPFACDICGRKFADRSNLTRHTKIHLRGSQLVKSELEEKKSELRHKLKYVPHEYIELIEIARNPTQDRILEMKVMEFFMKVYGYRGEHLGGSRKPDGAIYTVGSPIDYGVIVDTKAYSGGYNLPIGQADEMQRYVEENQTRNKHINPNEWWKVYPSSVTEFKFLFVSGHFKGNYKAQLTRLNHITNCNGAVLSVEELLIGGEMIKAGTLTLEEVRRKFNNGEINF

**>Three-NLS CXCR4 ZFN Right**

MGSSHHHHHHSSGLVPRGSHMPKKKRKVLEPKKKRKVPGMAPKKKRKVGIHGVPAAMAERPFQCRICMRNFSRSDSLLRHIRTHTGEKPFACDICGRKFARSDHLTTHTKIHTGSQKPFQCRICMRNFSRSDSLSAHIRTHTGEKPFACDICGRKFADRSNLTRHTKIHLRGSQLVKSELEEKKSELRHKLKYVPHEYIELIEIARNPTQDRILEMKVMEFFMKVYGYRGEHLGGSRKPDGAIYTVGSPIDYGVIVDTKAYSGGYNLPIGQADEMQRYVEENQTRNKHINPNEWWKVYPSSVTEFKFLFVSGHFKGNYKAQLTRLNHITNCNGAVLSVEELLIGGEMIKAGTLTLEEVRRKFNNGEINF

**>Four-NLS CXCR4 ZFN Right**

MGSSHHHHHHSSGLVPRGSHMPKKKRKVLEPKKKRKVGGSPKKKRKVPGMAPKKKRKVGIHGVPAAMAERPFQCRICMRNFSRSDSLLRHIRTHTGEKPFACDICGRKFARSDHLTTHTKIHTGSQKPFQCRICMRNFSRSDSLSAHIRTHTGEKPFACDICGRKFADRSNLTRHTKIHLRGSQLVKSELEEKKSELRHKLKYVPHEYIELIEIARNPTQDRILEMKVMEFFMKVYGYRGEHLGGSRKPDGAIYTVGSPIDYGVIVDTKAYSGGYNLPIGQADEMQRYVEENQTRNKHINPNEWWKVYPSSVTEFKFLFVSGHFKGNYKAQLTRLNHITNCNGAVLSVEELLIGGEMIKAGTLTLEEVRRKFNNGEINF

**Table S2.** Amino acid sequences of the ZFN proteins used in this study. Zinc-finger and FokI cleavage domains are colored orange and purple, respectively.
